# Effect of a virtual reality simulator for preclinical instruction of operative dentistry on level of competence of undergraduate dental students

**DOI:** 10.1186/s12909-025-08045-2

**Published:** 2025-10-21

**Authors:** Mohammad Mehdi Heidaridarani, Fatemeh Farzaneh, Gita Rezvani, Soleiman Ahmady, Fahimeh Dehestani Ardakani, Mohammad Hossein Mahrooz

**Affiliations:** 1 School of Dentistry, Shahed University, Tehran, Iran; 2 Department of Restorative Dentistry, School of Dentistry, Shahed University, Tehran, Iran; 3 Department of Oral Pathology, School of Dentistry, Shahed University, Tehran, Iran; 4 Department of Medical Education, School of Medical Education & Learning Technologies, Shahid Beheshti University of Medical Sciences, Tehran, Iran; 5 School of Medicine, Shahid Beheshti University of Medical Sciences, Tehran, Iran

**Keywords:** Education, dental, graduate, Virtual reality, High fidelity simulation training, Educational assessment, Clinical competence, Dentistry, operative

## Abstract

**Background:**

This study aimed to assess the effect of a virtual reality (VR) simulator for instruction of restorative dentistry on the level of competence of undergraduate dental students.

**Methods:**

This case-control study was conducted on 55 third-year dental students, who were randomly assigned to the intervention (*n* = 30) and control (*n* = 25) groups. Both groups received the same theoretical instruction for preparation of a Class I cavity in a mandibular first molar. The control group then practiced cavity preparation on acrylic teeth for 8 h. The intervention group practiced by using a VR simulator for 4 h and practicing on acrylic teeth for 4 h. Both groups participated in a pretest on acrylic teeth. The intervention group had one posttest after using the VR simulator and another posttest after practicing on acrylic teeth. The control group also had two posttests after the first and second phases of practice. The performance of the two groups was scored blindly by three examiners. Data were analyzed by independent and paired t-test, Chi-square test, Fisher’s exact test, and Mann-Whitney U test (alpha = 0.05).

**Results:**

The improvement in overall performance was significantly greater in the intervention group than the control group (*P* < 0.05). Progression in the first step (pretest-posttest 1) was significantly greater in 5 out of 9 criteria in the intervention group than the control group (*P* < 0.05). Progression in the second step (posttest 1-posttest 2) was significantly greater in the intervention group than the control group in 6 out of 9 criteria (*P* < 0.05). The reduction in catastrophic errors in the first posttest compared to pretest was significantly greater in the intervention group than the control group (*P* = 0.000 for the VR group and *P* = 0.006 for the control group).

**Conclusions:**

VR simulation can improve the quality of learning of preclinical restorative dentistry, and may be used as an educational supplement in dental curricula.

## Introduction

Simulation plays a key role in enhancing the quality of dental education by providing a controlled, risk-free environment for developing clinical skills. In dental training, simulators—whether physical models or virtual reality (VR) systems—bridge the gap between theoretical instruction and clinical practice, allowing students to improve their competence without exposing patients to potential harm.

Traditionally, preclinical dental education has relied on phantom heads and typodonts to teach foundational manual skills. Although these tools provide essential practice, VR simulators have shown superior outcomes by offering immersive, three-dimensional environments and real-time feedback. Evidence highlights the benefits of VR in teaching restorative dentistry, prosthodontics, and oral surgery, through technologies such as haptic feedback, augmented reality, and real-time digital mapping [1–4]. 

Ensuring patient safety and maintaining high standards of care are core ethical principles in dental education. Early clinical exposure without sufficient technical preparation may result in irreversible harm to patients, such as compromised tooth integrity or procedural complications [5, 6]. Therefore, preclinical training must offer a structured and safe environment where students can acquire and refine psychomotor skills prior to engaging with real patients. In this context, virtual reality (VR) simulators have emerged as ethically responsible tools that allow risk-free, effective learning [7, 8].

Conventional preclinical dental training, relying on typodonts and phantom heads, provides foundational practice but lacks immersive feedback and fails to replicate the complexity of clinical conditions [9, 10]. In contrast, VR simulators offer immersive, three-dimensional environments with real-time feedback, haptic interaction, and error-tracking features that enhance skill acquisition while supporting patient safety [11, 12]. VR also enables deliberate practice through repetition and feedback, aligning well with theories of motor learning [13, 14].

In addition to enhancing technical competence, VR technology contributes meaningfully to the cognitive and emotional dimensions of learning. Beyond manual training, VR and serious games have shown to boost student motivation, self-efficacy, and engagement—key drivers of effective learning in modern curricula [9]. This is particularly relevant in the post pandemic educational landscape, where technology-enhanced learning (TEL) has expanded rapidly, reshaping expectations for digital literacy in healthcare education [8, 15].

Despite these promising developments, the effectiveness of VR in dental education remains an active area of investigation, especially regarding its integration into conventional preclinical courses. Despite growing interest, there is still a need for empirical research comparing VR-integrated instruction with conventional training in specific preclinical tasks. This study aimed to evaluate the impact of the DentaSim VR simulator (SiMedix) on the competence of undergraduate dental students in performing Class I cavity preparation, assessing whether VR can enhance learning outcomes and reduce procedural errors when integrated into conventional dental curricula.

## Methods

This case-control study was conducted on 55 third-year undergraduate dental students enrolled in the preclinical restorative dentistry course.

### Ethical approval and consent to participate

The study protocol was approved by the Research Ethics Committee of Shahed University (IR.SHAHED.REC.1402.055). Written and verbal informed consent was obtained from all participants, following a protocol approved by the university’s ethics committee. This study also adheres to the ethical principles of the Declaration of Helsinki.

As the intervention was purely educational and conducted in a preclinical laboratory setting using simulation models (typodonts and a virtual reality simulator), no clinical procedures involving patients or health-related outcomes were performed. Therefore, according to the Iranian Registry of Clinical Trials (IRCT) guidelines and the WHO ICTRP criteria, clinical trial registration was not applicable for this study.

### Sample size calculation

The sample size for this case-control study was determined based on prior data from [16], who reported a mean improvement in clinical skills scores of 20.4% (from 65.3%, SD = 7.2% to 85.7%, SD = 6.1%) in the VR-based simulation group, compared to a 7.9% improvement (from 64.5%, SD = 6.8% to 72.4%, SD = 7.0%) in the control group. The estimated effect size (difference in mean improvement) was approximately 12.5%.

A pooled standard deviation of the change scores was calculated as 8.05%, assuming a correlation coefficient of 0.3 between pre- and post intervention scores. Using a two-sided α = 0.05 and power (1-β) = 90%, the minimum required sample size was calculated using standard formulas for two independent means, resulting in 9 participants per group (total 18). To account for an anticipated dropout rate of 10%, the sample size was adjusted to 10 participants per group (total 20). Although the initial sample size calculation indicated that 20 participants (10 per group) would be sufficient, the total number of available students in our setting was 55. To maximize participant inclusion and educational benefit, and to strengthen the generalizability of the findings, we decided to include all 55 students in the study.

Given that the total number of eligible students was 55, and considering the reported prevalence of VR-related side effects such as nausea, dizziness, and eyestrain (approximately 21% based on [17]) we anticipated a higher dropout risk in the VR group. Therefore, to maintain adequate statistical power in the presence of possible attrition, participants were allocated in a 5:6 ratio (control-VR). Ultimately, 25 participants were assigned to the control group and 30 participants to the VR group (total *N* = 55), providing >95% power to detect the target effect size even under conservative assumptions regarding attrition or variability.

### Randomization and allocation concealment

Participants were randomly assigned to either the VR group or the control group using a computer-generated randomization sequence. Block randomization (block sizes of 5 and 6) was applied to maintain approximately balanced group sizes throughout the enrollment period. Allocation concealment was ensured using sequentially numbered, opaque, sealed envelopes. At enrollment, the study coordinator opened the next envelope in sequence in the presence of the participant to reveal their assigned group. Participants who experienced significant VR-related side effects (nausea, dizziness, eyestrain), as defined in the exclusion criteria, were withdrawn from the training sessions to prevent adverse impacts on the learning process. Their data were excluded from the final analysis as per the predefined exclusion criteria.

### Intervention and control procedures

The control group conventionally practice on acrylic typodonts in the restorative dentistry preclinical course, and the intervention group use a VR simulator in addition to the conventional method of practice to prepare Class I amalgam cavities in mandibular first molars.

Prior to practical instruction, all students received theoretical instructions regarding the principles of amalgam Class I cavity preparation for small and large occlusal caries. A Class I cavity preparation according to SISTA classification on a mandibular first molar was instructed to students in the present study. The sessions and educational content in the two groups are presented in Table 1. As shown, the VR group received 4 h of conventional instruction and practice on typodonts plus 4 h of instruction and practice by using a VR simulator (DentaSim, SiMedix); while, the control group received 8 h of conventional instruction and practice on typodonts.

**Table 1:** Sessions and educational content in the two groups

Session	VR group	Control group
First session	-Presentation of preparation of basic geometrical shapes -Practicing preparation of basic geometrical shapes with the help of a mentor on a typodont (2 h)	-Presentation of preparation of basic geometrical shapes -Practicing preparation of basic geometrical shapes with the help of a mentor on a typodont (2 h)
Second session	-Presentation of Class I cavity preparation -Pretest -Practicing preparation of basic shapes in the simulator in absence of a mentor (2 h)	-Presentation of Class I cavity preparation -Pretest -Practicing preparation of basic shapes with the help of a mentor (2 h)
Third session	-Practicing a Class I cavity preparation in the simulator in absence of a mentor (2 h) -First posttest (posttest 1)	-Practicing a Class I cavity preparation and delivering the prepared sample with the help of a mentor (2 h) -First posttest (posttest 1)
Fourth session	-Practicing a Class I cavity preparation with the help of a mentor (2 h) -Second posttest (posttest 2)	-Practicing a Class I cavity preparation with the help of a mentor (2 h) -Second posttest (posttest 2)

**Fig. 1:**
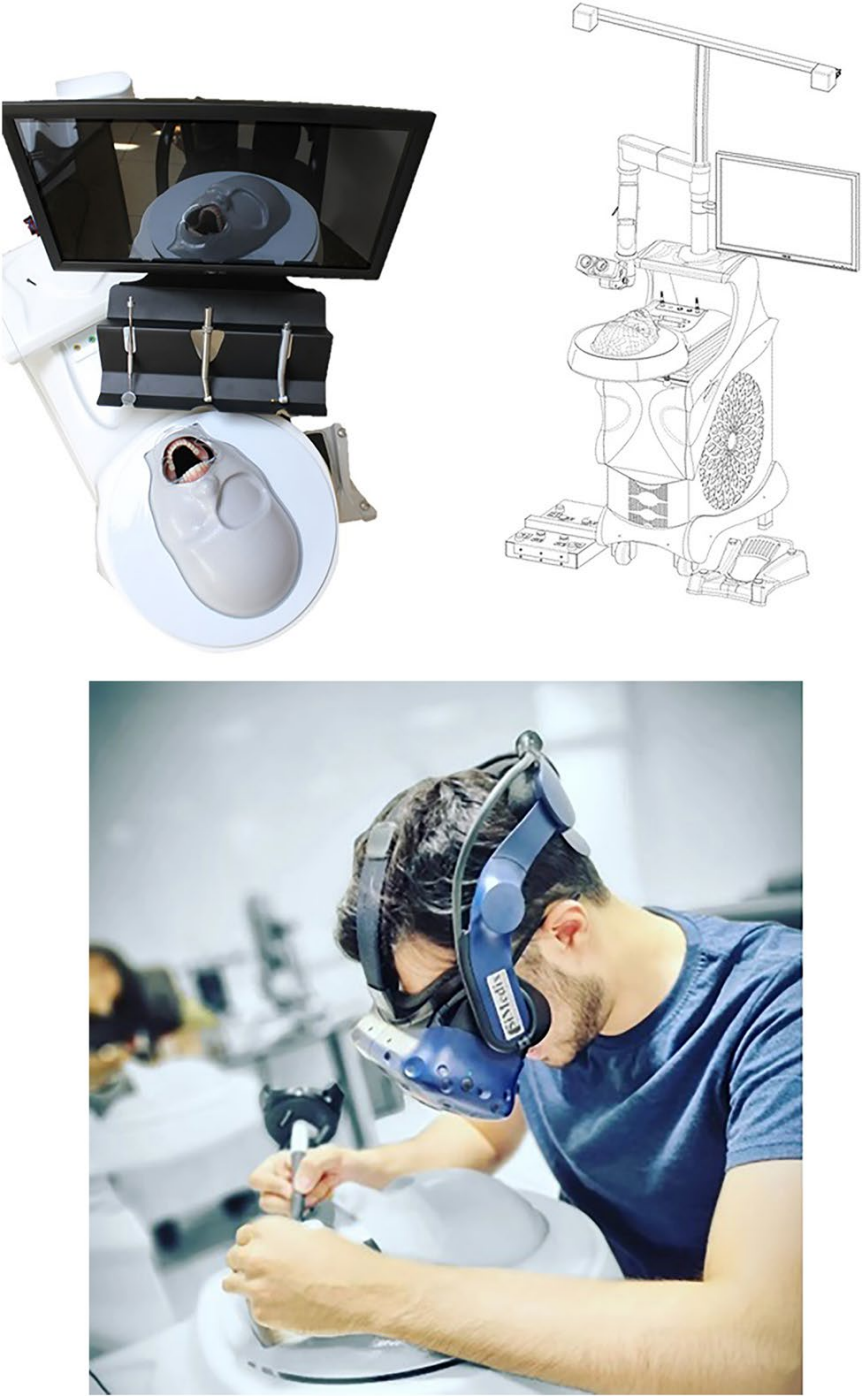
VR simulator used in the present study

**Fig. 2:**
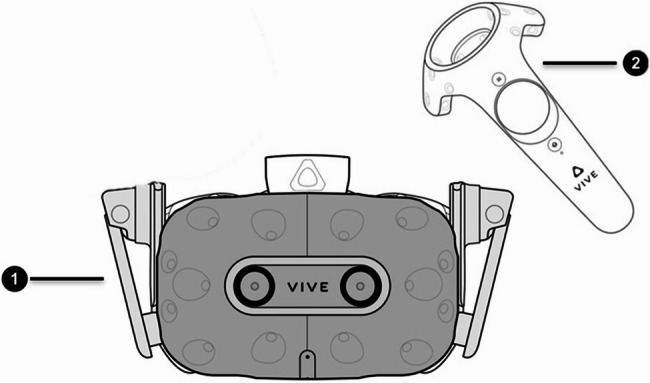
(1) HMD (Head Mounted Display) and (2) controller, of DentaSim VR simulator

Figure 1 shows the VR simulator used in the present study. In this simulator, a movement recording system records the hand movements of the operator with 20 μm accuracy, and transfers the data to a computer. The computer processes the performance of the operator according to predesigned equations and a graphic processor presents the results as a graphical render, which is displayed for the operator through the VR headset of the simulator. The main components of the simulator’s user interface, including the headset and the controller, are shown in Fig. 2.

Both groups participated in one pretest at the onset of the experiment, and two posttests at the end of the first and second phases of the instruction and practice. In these tests, the students were asked to prepare a Class I cavity on an acrylic typodont.

### Design and validation of the data collection tool

The data collection tool used in this study was a structured checklist designed to evaluate Class I amalgam cavity preparations on typodonts. The initial draft of the checklist was developed based on the educational content and procedural standards presented in [18].

To ensure content validity, the checklist was reviewed by four faculty members (mentors) from the Department of Restorative Dentistry at Shahed Dental School. These faculty members were experts in operative dentistry but were not involved in the scoring process. They evaluated the checklist for comprehensiveness, clarity, clinical relevance, and alignment with educational objectives. Based on their feedback, revisions were made to ensure the checklist accurately represented the critical components of cavity preparation.

Face validity was assessed by pilot testing the checklist with a small group of dental students who were not part of the main study sample. Students and faculty provided feedback on the clarity and applicability of each item, leading to further refinement of the tool prior to its final use.

### Scoring system and criterion weighting

The final checklist comprised multiple criteria reflecting both psychomotor and cognitive skills required for high-quality cavity preparation. Each criterion was scored on a 0–5 scale. To reflect the clinical significance of each criterion, weights were assigned to each item after consultation with four senior mentors in restorative dentistry (Table 2).

**Table 2:** Weighing of the criteria according to the opinion of the mentors (each criterion was weighed from 1 to 3)

Number	Criteria	Mentor 1	Mentor 2	Mentor 3	Mentor 4	Mean weight
1	Taper of mesial and distal walls	2	1	2	1	1.5
2	Cavity pulpal depth	1	3	2	3	2.25
3	Convergence of buccal and lingual walls	1	1	1	1	1
4	Perpendicular position of buccal and lingual walls relative to external tooth surface	1	1	1	1	1
5	Outline form following the shape of grooves with no sharpness	1	1	2	1	1.25
6	Outline: Following the cuspal form	2	1	1	1	1.25
7	Round and distinct line-angles	1	1	2	1	1.25
8	Proper distance from the mesial and distal marginal ridges	2	2	2	2	2
9	Sufficient isthmus and cavity width	1	2	1	2	1.5

The overall competency score for each student represented the quality of their cavity preparation and served as a proxy for foundational clinical competence. This score integrated technical skills (e.g., bur angulation control, pulpal depth accuracy) and cognitive understanding of the ideal cavity form and its rationale.

### Critical criteria definition

In addition, based on the mentors’ consensus, certain criteria were designated as critical: Adequate pulpal floor depth, Proper distance from mesial and distal marginal ridges and Appropriate isthmus and cavity width. If a student failed to achieve an acceptable score on any of these critical criteria, their overall performance was considered unacceptable, regardless of the overall score.

### Scoring procedure and blinding

Three restorative dentistry faculty members from Shahed Dental School, with extensive experience in cavity preparation, independently assessed the cavity preparations. All assessors were blinded to the students’ identities and the type of test (pretest, posttest 1 or posttest 2) to reduce potential bias. Each cavity preparation was scored by each assessor on three separate occasions to assess intra-rater consistency. The final score for each cavity was the mean of the three scores given by the same assessor.

### Reliability assessment

Intra-rater reliability: For each assessor, Cronbach’s alpha was calculated across their three scoring rounds to measure the consistency of their evaluations. Inter-rater reliability: Cronbach’s alpha was also calculated across the average scores of the three assessors to assess the agreement between different raters.

### Measures to minimize contamination bias

To prevent contamination bias between the intervention and control groups:

- The intervention group practiced on simulators located outside the preclinical laboratory, separated from the area where the control group trained.
- The study period was intentionally short to limit interactions between the two groups.
- Students were explicitly instructed not to engage in extracurricular practice or share their learning experiences with peers during the study period [19, 20].

### Additional evaluation parameters

In addition to the overall competency score, the quality of cavity preparation was evaluated in terms of spatial parameters (mesiodistal divergence, pulpal depth, buccolingual convergence, buccolingual perpendicularity) and non-spatial parameters (fissure outline, cusp outline, line angles, marginal ridge distance, and cavity width).

A detailed explanation of the scoring criteria, clinical criticality, and dependency on spatial cognition is presented in Table 3.

**Table 3:** Categorization of evaluating criteria based on clinical importance and Spatial cognition relevance

Number	Criteria	Description	Clinical criticality	Spatial Cognition
1	MD divergence	Taper of mesial and distal walls	No	Yes
2	pulpal depth	Cavity pulpal depth	Yes	Yes
3	BL Convergence	Convergence of buccal and lingual walls	No	Yes
4	BL-perpendicularity	Perpendicular position of buccal and lingual walls relative to external tooth surface	No	Yes
5	Fissure outline	Outline form following the shape of grooves with no sharpness	No	No
6	Cusp outline	Outline: Following the cuspal form	No	No
7	Line angle	Round and distinct line-angles	No	No
8	MR distance	Proper distance from the mesial and distal marginal ridges	Yes	No
9	Width	Sufficient isthmus and cavity width	Yes	No

The following exercises were considered for students in the VR group:

- Exercise for hand tremor: This exercise measured the hand tremor during vertical movements to correct it. This exercise is useful for restorative cavity, access cavity, and prosthetic tooth preparations.
- Preparation of simple geometrical shapes: Considering the complexity of different preparations on actual teeth, the students should learn the principles of preparation of cavities and simple shapes, and practice prior to starting more complex preparations. In this exercise, simple geometrical shapes were prepared. The geometrical shapes were designed in an attempt to prepare the students for more complex preparations in the next steps.
- Preparation of simple patterns: This exercise instructed the students on how to prepare the required patterns such as observing the correct depth of preparation, taper of the walls (divergence/convergence), etc. Another exercise focused only on the correct angulation of the walls. Focusing only on this parameter with no concern regarding occlusal form or depth improved students’ attention to angulation of the grip and improved the quality of learning through micro-learning.
- Preparation of the outline form: This exercise improved the students’ attention to the occlusal shape of the cavity with no concern regarding other geometrical parameters in a standard preparation. Preparation of a 2D tooth model helps the students to have a better understanding of the occlusal form.
- Guided preparation: In this exercise, cavity preparation was performed by using a guide. Students had to pay attention to the correct pattern shown by the guide during cavity preparation. This exercise would greatly help in correct understanding of the cavity form.
- Complete preparation: In this exercise, the students were provided with a complete tooth with enamel, dentin, and pulp and they could select any preparation design for it. The students could save the results at the end of the exercise, and this file would also be saved in the mentor’s profile as the final performance of student. The mentor could observe it, score it, and provide feedback.
- Supplemental tools: The VR technology can provide numerous features. For instance, the students could enable the transparency feature to see a transparent view of the tooth to assess the relationship of the prepared cavity with the dental pulp and prevent iatrogenic mechanical exposure of the pulp. Also, the students could assess the flatness of the cavity floor easily. Magnification would also help in better assessment of the details of preparation. Furthermore, the students could superimpose the final prepared cavity on intact tooth to see the degree of removal of the tooth structure.

### Statistical analysis

For each continuous variable, the assumption of normality within each randomized group was assessed using the Kolmogorov–Smirnov test and by visually inspecting Q–Q plots. When the Kolmogorov–Smirnov test did not reject normality (*p* > 0.05) and the Q–Q plots demonstrated approximate linearity, the independent samples t-test was used to compare groups. If normality was rejected (*p* < 0.05) or Q–Q plots showed clear deviations from linearity, the non-parametric Mann–Whitney U test was applied.

Demographic variables were compared using the independent samples t-test for continuous variables and the Chi-square test or Fisher’s exact test for categorical variables. Within-group changes in test scores were assessed using the paired t-test. All statistical analyses were performed using SPSS version 26 (IBM Corp., Somers, NY, USA), and a two-tailed p-value < 0.05 was considered statistically significant.

## Results

The inter-rater reliability value was found to be 0.786 indicating good inter-rater reliability, and the intra-rater reliability values were all >0.940, indicating excellent intra-rater reliability for all three assessors [21]. Of a total of 55 students, 15 were females (27%) and 40 were males (73%). The Chi-square test showed no significant difference in gender distribution between the two groups (*P* = 0.619). Table 4 presents the measures of central dispersion for the criteria scores and pretest and posttest scores acquired by the two groups. The two groups had no significant difference in the pretest score (*P* = 0.246).

**Table 4:** Descriptive statistics (mean ± SD) for the criteria scores and pretest and posttest scores in the two groups^a^

Test	Parameter	Control group (*N* = 25)	VR group (*N* = 30)
Pretest	MD-divergence	2.17 ± 0.71	2.03 ± 0.99
	Pulpal depth	2.15 ± 1.00	1.90 ± 1.06
	BL-convergence	2.34 ± 0.83	2.12 ± 1.04
	BL-perpendicularity	2.30 ± 0.85	2.10 ± 0.95
	Fissure outline	1.92 ± 0.81	1.60 ± 0.85
	Cusp outline	2.10 ± 0.93	1.78 ± 1.02
	Line angle	1.75 ± 0.77	1.62 ± 0.95
	MR distance	2.52 ± 0.81	2.08 ± 0.96
	Width	2.40 ± 0.82	2.21 ± 1.03
	Overall	2.20 ± 0.71	1.94 ± 0.88
Posttest 1	MD-divergence	2.70 ± 0.71	3.03 ± 0.78
	Pulpal depth	2.66 ± 0.76	3.11 ± 0.81
	BL-convergence	2.81 ± 0.70	3.23 ± 0.83
	BL-perpendicularity	2.91 ± 0.71	3.24 ± 0.80
	Fissure outline	2.54 ± 0.92	2.71 ± 0.94
	Cusp outline	2.69 ± 0.98	2.79 ± 0.90
	Line angle	2.42 ± 0.86	2.61 ± 0.95
	MR distance	2.91 ± 0.82	2.91 ± 0.94
	Width	2.84 ± 0.92	2.97 ± 1.01
	Overall	2.72 ± 0.72	2.96 ± 0.78
Posttest 2	MD-divergence	2.89 ± 0.74	3.71 ± 0.54
	Pulpal depth	2.93 ± 0.83	3.66 ± 0.73
	BL-convergence	3.04 ± 0.76	3.80 ± 0.71
	BL-perpendicularity	3.10 ± 0.72	3.80 ± 0.66
	Fissure outline	2.81 ± 0.92	3.52 ± 0.78
	Cusp outline	2.92 ± 0.87	3.62 ± 0.75
	Line angle	2.55 ± 0.99	3.24 ± 0.78
	MR distance	3.10 ± 0.83	3.76 ± 0.66
	Width	3.15 ± 0.86	3.73 ± 0.77
	Overall	2.95 ± 0.72	3.65 ± 0.59

^a^Full statistical details (including 95% CIs) are available upon request

### Within-group comparisons

As shown in Table 5, the first phase (pretest-posttest 1) was significantly effective in both groups (*P* < 0.05). In the second phase (posttest 1-posttest 2), no significant improvement was observed in criteria #1 and criteria #3 to #8 in the control group (*P* > 0.05) while all criteria significantly improved in the VR group in the second phase (*P* < 0.05). The difference in posttest 1 and posttest 2 was not significant in the control group (*P* < 0.05). As previously discussed in the explanation of the “Overall” score, this metric encompasses both manual dexterity and cognitive insight (two core components of clinical competence). Therefore, an improvement is not merely a statistical gain, but rather an indication of enhanced readiness for real-world clinical performance. This reinforces the clinical relevance of the observed improvement.

**Table 5:** Comparison of pretest and posttest scores acquired by students in the two groups in each criterion by paired samples t-test

Group	Criteria	Pretest-posttest 1		Posttest 1-posttest 2	
		Within-group mean difference ± SD^a^	*P* value	Within-group mean difference ± SD^a^	*P* value
Control group	MD-divergence	0.53 ± 0.63	**0.000**	0.20 ± 0.53	0.079
	Pulpal depth	0.51 ± 1.21	**0.046**	0.27 ± 0.58	**0.029**
	BL-convergence	0.47 ± 0.73	**0.004**	0.22 ± 0.57	0.065
	BL-perpendicularity	0.61 ± 0.91	**0.003**	0.19 ± 0.50	0.067
	Fissure outline	0.62 ± 0.94	**0.003**	0.27 ± 0.73	0.080
	Cusp outline	0.59 ± 1.04	**0.009**	0.23 ± 0.77	0.153
	Line angle	0.67 ± 0.82	**0.000**	0.12 ± 0.66	0.357
	MR distance	0.38 ± 0.64	**0.006**	0.19 ± 0.76	0.223
	Width	0.44 ± 1.02	**0.040**	0.30 ± 0.60	**0.020**
	Overall	0.52 ± 0.66	**0.001**	0.22 ± 0.47	**0.024**
VR group	MD-divergence	1.01 ± 0.94	**0.000**	0.68 ± 0.59	**0.000**
	Pulpal depth	1.21 ± 1.06	**0.000**	0.55 ± 0.84	**0.001**
	BL-convergence	1.11 ± 0.91	**0.000**	0.56 ± 0.69	**0.000**
	BL-perpendicularity	1.14 ± 0.88	**0.000**	0.55 ± 0.56	**0.000**
	Fissure outline	1.11 ± 0.98	**0.000**	0.81 ± 0.88	**0.000**
	Cusp outline	1.01 ± 1.00	**0.000**	0.84 ± 0.83	**0.000**
	Line angle	0.99 ± 1.03	**0.000**	0.63 ± 0.86	**0.000**
	MR distance	0.83 ± 0.97	**0.000**	0.85 ± 0.83	**0.000**
	Width	0.76 ± 0.98	**0.000**	0.75 ± 0.98	**0.000**
	Overall	1.01 ± 0.81	**0.000**	0.70 ± 0.60	**0.000**

*SD* Standard deviation, *BL* Buccolingual, *MD* Mesiodistal, *MR* Marginal ridge

^a^Negative values indicate greater improvement in the VR group

Bold values indicate statistically significant *p*-values (*p* < 0.05)

### Between-group comparisons

Comparison of the two groups regarding each criterion in the first and second phase of instruction (Table 6) revealed that in the first phase, the VR group had a significantly superior performance in 5 out of 9 criteria (*P* < 0.05). In the second phase, the VR group had a significantly superior performance in 6 out of 9 criteria compared to the control group (*P* < 0.05). The two groups had no significant difference in the pretest score (*P* = 0.246) and posttest 1 score (*P* = 0.259). However, the VR group acquired a significantly higher score in the posttest 2 than the control group (*P* = 0.000).

**Table 6:** Group comparisons across instructional phases: by independent samples t-test and VR superiority in spatial/non-spatial

Spatial cognition	Criteria	Pretest-posttest 1		Posttest 1-posttest 2	
		Mean difference	*P* value	Mean difference	*P* value
Spatial	MD-divergence	−0.479	**0.029**	−0.486	**0.002**
	Pulpal depth	−0.696	**0.029**	−0.277	0.155
	BL-convergence	−0.643	**0.005**	−0.341	0.051
	BL-perpendicularity	−0.530	**0.034**	−0.361	**0.014**
Non-spatial	Fissure outline	−0.489	0.065	−0.540	**0.016**
	Cusp outline	−0.416	0.138	−0.610	**0.007**
	Line angle	−0.317	0.210	−0.506	**0.017**
	MR distance	−0.318	0.248	−0.657	**0.004**
	Width	−0.451	**0.043**	−0.450	0.050
	Overall	−0.490	**0.019**	−0.470	**0.002**

Bold values indicate statistically significant *p*-values (*p* < 0.05)

### Spatial and non-spatial parameters

As indicated in Table 6, the VR group was superior to the control group in all spatial parameters and one non-spatial parameter (width) in the first phase (*P* < 0.05), and two spatial parameters and four non-spatial parameters in the second phase (*P* < 0.05).

### Final acceptance

As shown in Table 7, the percentage of acceptance was almost the same in the two groups in the pretest (*P* = 0.665). However, in the second phase, the VR group had a significant superior performance in terms of acceptance percentage (*P* = 0.004). No significant effect of practice for more than 4 h on the performance of the control group was evident (*P* > 0.05).

**Table 7:** Percentage of acceptance and frequency of critical errors

Variable	Test	VR group	Control group
		Mean ± SD	Mean ± SD
Percentage of acceptance	Pretest	7%	4%
	Posttest 1	43%	40%
	Posttest 2	67%	28%
Critical errors	Pretest	2.33 ± 0.98	2.20 ± 0.89
	Posttest 1	1.20 ± 1.22	1.40 ± 1.33
	Posttest 2	0.50 ± 0.92	1.40 ± 1.17

### Critical errors

As shown in Table 7, the mean frequency of critical errors significantly decreased in the VR group during the process of instruction (*P* = 0.000 for the first phase and *P* = 0.004 for the second phase); while it had no significant change in the second phase of instruction in the control group (*P* = 0.006 for the first phase and *P* = 1.000 for the second phase). Also, VR instruction had a significantly greater effect on reduction of critical errors in the second phase (*P* = 0.002).

## Discussion

This study assessed the effect of a VR simulator for instruction of restorative dentistry on the level of competence of undergraduate dental students. The results showed the superiority of VR group in overall educational progression throughout the experiment, which was in agreement with previous results regarding optimal efficacy of VR simulators for improvement of educational quality and efficiency [13, 16, 22, 23].

The VR group showed significant improvement in 6 out of 9 tested criteria while no significant improvement was found in the control group in the second phase of the instruction (posttest 1-posttest 2). This finding indicates that the conventional learning through practicing on acrylic typodonts has a plateau, which was reached after 4 h of practice in the present study; when reached, further practice would not cause any significant improvement in learning. This decline in performance growth in the control group became evident in posttest 2 and appears to be associated with the absence of feedback during their training process. The lack of appropriate feedback during practice can lead to a premature plateau in performance, known as “arrested development.” Although this level exceeds everyday skill, it falls short of expert performance [24].

In various fields of skill training (including psychotherapy [25], resuscitation training [26], and clinical education [27]) learners engaging solely in standard or repetitive practice often reach a plateau after initial improvements, a phenomenon described as arrested development [24].

This stagnation can only be overcome through structured, feedback-rich deliberate practice. These findings are consistent with our results, where participants in the control group failed to demonstrate further improvement beyond a certain point, indicating a similar plateau in learning progression [28]. This phenomenon has been consistently observed in the literature on sports science and music, and it is likely to have a comparable impact on the development and retention of surgical skills [29].

Also, the results showed greater effect of VR simulator on spatial parameters, which indicates that VR simulator features can be of great help in learning spatial topics; whereas, the conventional method is sufficiently effective for learning of non-spatial parameters. The frequency of critical errors decreased in the VR group over time while no change in this regard was observed in the conventional group in the second phase of learning, pointing to saturation of educational quality after 4 h of conventional practice. When an acceptable level of competence is reached, further improvement is not often achieved by continuation of practice, and requires more advanced strategies [30–32]. Thus, to further improve the quality of conventional instruction, the student-mentor communication should be strengthened and new feedback strategies should be adopted; increasing the practice time alone with no additional feedback from mentors would not probably cause further improvement in this phase, and formative assessment is required during deliberate practice [33, 34]. However, in instruction with VR simulation, increasing the practice time may be helpful according to the theories of deliberate practice since VR simulators enable concentrated practice, self-assessment, feedback, and retry. The optimal efficacy of this method has been previously documented since simulation-based education in medicine aims to transfer the skills acquired through simulated practice to clinical practice [34]. In total, the present results confirmed the prominent role of VR simulator in improvement of the competence of dental students in restorative dentistry preclinical course, which was in line with previous findings [3, 35, 36].

Using VR simulators for enhancement of instruction can save the time of mentors and considerably decrease the need for constant presence of instructors [37, 38]. It is highly valuable considering the shortage of faculty members in dental and medical schools [12, 39].

Another advantage of using VR simulators is that they enable deliberate practice, and provide fast continuous feedback during the process, which are imperative for learning especially during the preclinical courses. In conventional instruction, students often receive feedback after completion of their practice while VR simulators provide feedback at each step of the process, which improves the quality of self-assessment and self-correction by students [40–43]. Several studies have shown the effective role of advanced simulators such as VR simulators in enhancement of the quality of education in healthcare fields [44–47]. Nonetheless, some others reported that simulation-based education in medicine did not significantly improve the competence of students [48] possibly because the training was limited to a brief single session, which had little observable impact when followed by six months of real-life clinical experience.

Micro learning is another advantage of using VR simulators for instruction. During a tooth preparation process, a high number of parameters and principles should be followed for an acceptable outcome. In the conventional education, it is often difficult for students to pay attention all these parameters at the same time; however, VR simulators use the micro learning method and break down the educational content into smaller parts for presentation and practice, which accelerates and enhances the learning process, and improves knowledge retention [15].

Deliberate learning has been suggested for learning of skills that require a higher level of cognitive load and are less likely to be learned through rote muscle memory such as point of care ultrasound [49], ultrasound-guided regional anesthesia [50], and team work practice during simulated resuscitation of children [51]. In dentistry, such skills include restorative cavity preparation, endodontic access cavity preparation, crown preparation, and implant osteotomy site preparation, among others. Furthermore, VR simulation has a higher degree of compliance with the reality compared with other electronic educational methods [52].

In the systematic review by [8], the effectiveness of virtual reality in dental anatomy education was reported to be only moderately positive, especially when compared to training with real patients or high-fidelity physical models. However, this apparent contradiction with the findings of our study likely arises from fundamental differences in the comparison conditions and the context of the training. While anatomy education can feasibly involve real clinical models or patients due to its observational nature, such resources are neither practical nor ethically justifiable for repetitive training in interventional procedures like restorative dentistry or endodontics. In contrast, our study compared VR-based training to conventional methods such as acrylic tooth models and lecture-based instruction, where VR demonstrated a clear educational advantage. Thus, the perceived inconsistency is more reflective of differing baselines rather than an inherent limitation of VR.

Although the present study demonstrates a positive effect of virtual reality simulation on preclinical cavity preparation training in restorative dentistry, the systematic review by [53] indicates that the impact of this technology on prosthodontic education is less significant. This discrepancy may be attributed to differences in the clinical focus (restorative versus prosthodontics), the type of VR simulators used, the experience level of participants, and the methods of performance assessment. However, the positive findings reported in implant surgery and restorative cavity preparation within the systematic review align with the current study’s results, suggesting that the effectiveness of VR technology in clinical education may depend on the specific skill set and educational context.

To better understand the implications of the present study, it is useful to compare virtual reality (VR)–based interventions with other commonly employed educational methods, such as simulated participants (SP) and serious games. For a comprehensive comparison, it is important to examine these methods across different domains, including knowledge and technical skills, communication skills, learners’ motivation and engagement and Cost and Scalability.

studies have reported the effectiveness of immersive and game-based educational interventions in enhancing knowledge and technical skills. Several systematic reviews and meta-analyses, including [54–56], have demonstrated positive effects of VR-based training methods and serious games on knowledge acquisition, skills development, and practical performance. In the domain of communication skills and professional behaviors, multiple studies support the effectiveness of SP-based methods in developing patient-centered communication skills [57, 58]. Moreover, studies have indicated that the effectiveness of educational methods using virtual reality (VR) and serious games is being actively investigated, showing promising results for enhancing communication skills and empathy in healthcare training [59, 60]. With regard to motivation and engagement, studies have reported that VR and serious games generally increase learners’ interest and active participation [61]. In terms of cost and scalability, VR and serious games often require high initial investment but offer greater scalability once developed [62]. In contrast, role-playing methods involve lower initial costs but lack comparable scalability.

Limitation: This study had some limitations. The faculty members were not familiar with the VR technology. This may have influenced the mentoring process and the effectiveness of feedback provided during training. we attempted to mitigate this by offering faculty orientation and relying on system-generated feedback, Also, due to differences with the conventional educational method, the mentors had to be trained in this regard [63]. Nonetheless, the VR simulator was well accepted by students since the new generation are all technology fans. Another limitation was that the simulated VR does not have 100% fidelity with the reality, which causes some concerns for the experienced mentors and faculty members. However, this problem can be obviated by further studies regarding the efficacy of VR simulators. Lack of educational standardization and diversity of VR simulators available in the market is another problem, which limits the generalization of the results obtained by using a specific type of VR simulator to other types [64]. In this study, we were only able to utilize the DentaSim simulator due to resource limitations. However, future studies are recommended to include a broader variety of virtual reality simulators to enhance the generalizability of findings across different systems and technologies.

The relatively short duration of the study limited our ability to assess long-term retention and transfer of skills from the simulator to real clinical settings. Although the results demonstrated immediate educational benefit. Since VR simulation represents a novel and engaging learning method, students in the VR group may have been more motivated or focused during the study, knowing they were being observed or using new technology. This could have positively influenced their performance, independent of the actual instructional advantage of VR. When evaluating the outcomes of educational interventions involving novel technologies such as virtual simulation, it is important to consider the potential influence of the Hawthorne Effect, as well as the inherent motivational boost that such innovations may provide, both of which can independently enhance learner performance [65]. Although the Hawthorne Effect may have contributed to greater motivation in the VR group, it also highlights VR’s capacity to actively engage learners—an important advantage in educational settings. Decreased face-to-face student-mentor communication, learning challenges, attitude of the users, and high cost are among other challenges of VR technology [66, 67].

Future research should explore several important directions to further enhance the integration and effectiveness of virtual reality in dental education. Studies are encouraged to evaluate the long-term retention and clinical transfer of skills acquired through VR training over extended follow-up periods. Comparing multiple VR platforms with varying technical and pedagogical features can help assess the consistency of educational outcomes across different systems. The sequence of VR instruction within the curriculum—whether implemented before or after conventional training—should also be investigated. In addition, expanding the use of VR to other clinical procedures such as root canal therapy, crown preparation, and implant placement can broaden its educational utility. Finally, comparative studies on instructional designs rooted in educational theories like microlearning, deliberate practice, and mastery-based learning within VR environments will provide deeper insights into optimizing learning efficiency and student engagement.

## Conclusion

VR simulation can improve the quality of learning of preclinical restorative dentistry and may be used as an educational supplement in dental curricula.

## Data Availability

The data used to support the findings of this study were supplied by corresponding author under license and data will be available on request. Requests for access to these data should be made to corresponding author

## Abbreviations

VR: Virtual Reality
HMD: head mounted display
